# Revealing the role of SPP1^+^ macrophages in glioma prognosis and therapeutic targeting by investigating tumor-associated macrophage landscape in grade 2 and 3 gliomas

**DOI:** 10.1186/s13578-024-01218-4

**Published:** 2024-03-21

**Authors:** Wenshu Tang, Cario W. S. Lo, Wei Ma, Annie T. W. Chu, Amy H. Y. Tong, Brian H. Y. Chung

**Affiliations:** 1 Hong Kong Genome Institute, 2/F, Building 20E, Hong Kong Science Park, Hong Kong, China; 2 Department of Pediatrics and Adolescent Medicine, School of Clinical Medicine, LKS Faculty of Medicine, The University of Hong Kong, Hong Kong, China

**Keywords:** Glioma, Tumor-associated macrophages, TAM-SPP1, *EGFR*, Immune suppression

## Abstract

**Background:**

Glioma is a highly heterogeneous brain tumor categorized into World Health Organization (WHO) grades 1–4 based on its malignancy. The suppressive immune microenvironment of glioma contributes significantly to unfavourable patient outcomes. However, the cellular composition and their complex interplays within the glioma environment remain poorly understood, and reliable prognostic markers remain elusive. Therefore, in-depth exploration of the tumor microenvironment (TME) and identification of predictive markers are crucial for improving the clinical management of glioma patients.

**Results:**

Our analysis of single-cell RNA-sequencing data from glioma samples unveiled the immunosuppressive role of tumor-associated macrophages (TAMs), mediated through intricate interactions with tumor cells and lymphocytes. We also discovered the heterogeneity within TAMs, among which a group of suppressive TAMs named TAM-SPP1 demonstrated a significant association with Epidermal Growth Factor Receptor (*EGFR*) amplification, impaired T cell response and unfavourable patient survival outcomes. Furthermore, by leveraging genomic and transcriptomic data from The Cancer Genome Atlas (TCGA) dataset, two distinct molecular subtypes with a different constitution of TAMs, *EGFR* status and clinical outcomes were identified. Exploiting the molecular differences between these two subtypes, we developed a four-gene-based prognostic model. This model displayed strong associations with an elevated level of suppressive TAMs and could be used to predict anti-tumor immune response and prognosis in glioma patients.

**Conclusion:**

Our findings illuminated the molecular and cellular mechanisms that shape the immunosuppressive microenvironment in gliomas, providing novel insights into potential therapeutic targets. Furthermore, the developed prognostic model holds promise for predicting immunotherapy response and assisting in more precise risk stratification for glioma patients.

**Graphical abstract:**

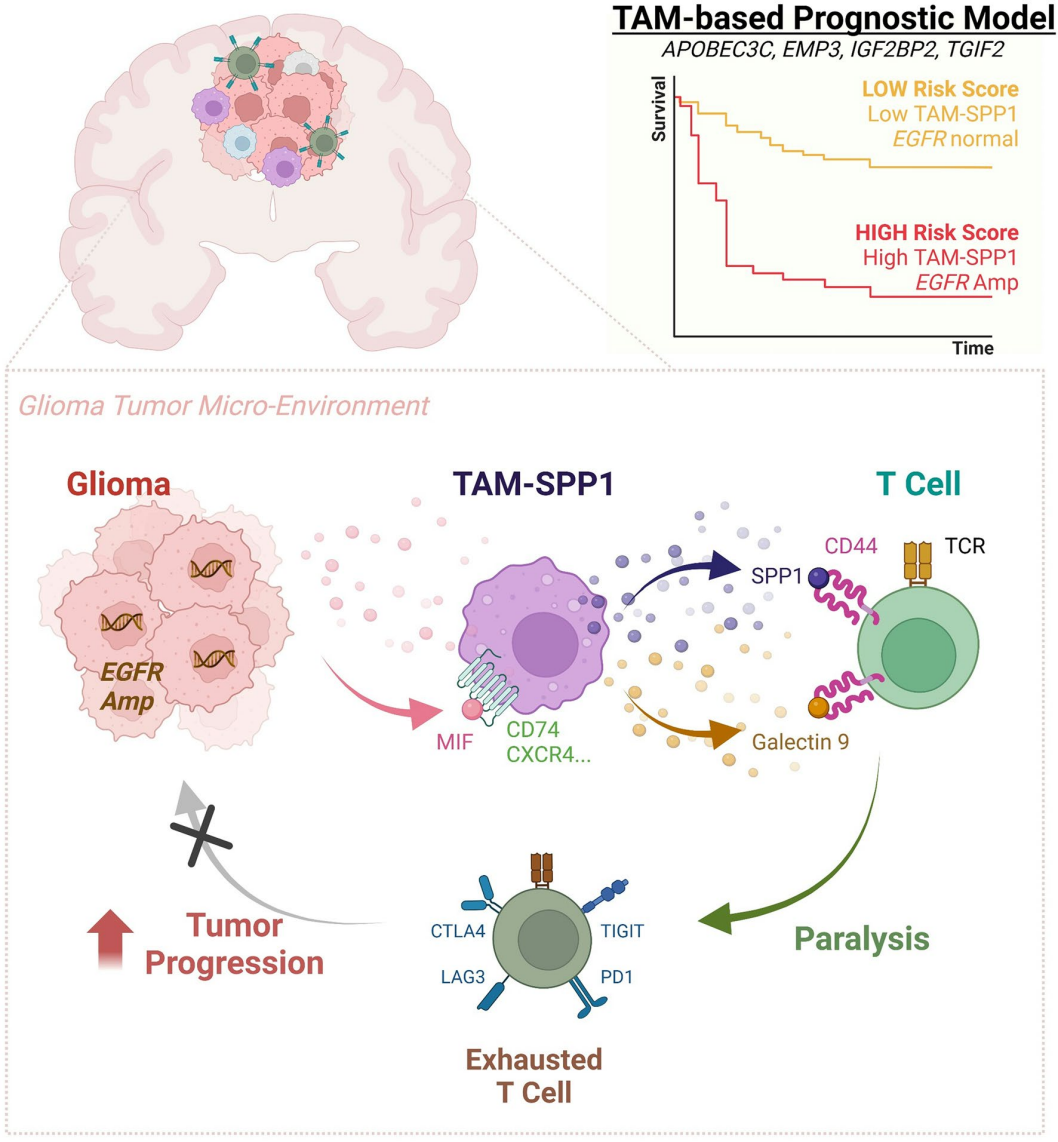

**Supplementary Information:**

The online version contains supplementary material available at 10.1186/s13578-024-01218-4.

## Introduction

Gliomas represent over 30% of primary brain tumors within the Chinese population and are one of the leading causes of cancer-related deaths globally [1, 2]. Gliomas exhibit remarkable heterogeneity, shaped by various genetic and epigenetic drivers as well as cellular activities [3–5]. The World Health Organization (WHO) classification of central nervous system tumors categorizes gliomas into grade 1–4 based on histopathological and molecular characteristics [5, 6]. Together, grade 2 and grade 3 gliomas account for over 30% of all glioma cases [7]. Despite being less aggressive than grade 4 glioblastoma (GBM)—which develops rapidly de novo with a median overall survival of merely 15 months [8]—grade 2 and 3 gliomas present a broad range of clinical behaviors and survival rates. The progression times for these gliomas can vary significantly, ranging from as short as two years to well over a decade [4, 9–11]. While genetic alterations such as *TP53*, isocitrate dehydrogenase (*IDH*), cyclin dependent kinase inhibitor 2A (*CDKN2A*) mutations and chromosome *1p/19q* codeletion are recognized contributors to glioma pathology, the importance of non-genetic factors in tumor progression and patient risk assessment is increasingly acknowledged [12]. Since current patient risk stratification relies solely on well-established genetic changes, it is imperative to identify key molecular factors that can enhance the precision of risk assessments in grade 2 and grade 3 gliomas.

Currently, the standard treatment options for gliomas are limited to surgical resection and chemoradiotherapy [7]. However, complete resection often poses a significant challenge, contributing to tumor relapse and progression. Moreover, residual tumor cells adapt to the immunosuppressive tumor microenvironment (TME), contributing to disease recurrence [13]. The glioma TME is a highly immunosuppressive milieu, posing a significant barrier to eradicating cancer cells and inducing antitumor immunity [14]. Therefore, re-writing the suppressive environment and boosting the patient’s own anti-tumor immune response is vital for favorable prognosis.

Researchers have uncovered the intricate interactions between glioma cells, neurons and immune cells, which support glioma progression and contribute to therapy resistance [15, 16]. Tumor-associated macrophages (TAMs), comprising both brain-resident microglia cells and bone marrow-derived macrophages, are recruited by tumor-derived cytokines and chemokines such as CXC motif chemokine ligand 16 (CXCL16), CC motif chemokine ligand 2 (CCL2), transforming growth factor beta (TGF-β) and interleukin 33 (IL-33) [17]. TAMs play crucial roles in therapy resistance by promoting cancer cell survival, inducing angiogenesis and suppressing CD8^+^T cell function through cell–cell interactions, suppressive cytokine secretion, and immune checkpoint upregulation [18–20]. Recent study suggests that TAMs initiate a prolonged immune response that results in T cell exhaustion and impedes antitumor immunity in glioblastoma [21]. Clinical attempts to disrupt the total TAM population by inhibiting the interactions between colony stimulating factor 1 receptor (CSF1R) with its ligands CSF1 and IL-34 have shown limited efficacy in GBM and other solid tumors [22, 23]. This ineffectiveness may be due to the compensatory activation or recruitment of other immune suppressive cells [24]. Therefore, precisely targeting specific macrophage subpopulations could improve therapy efficacy.

Advances in single-cell sequencing technology have revealed transcriptomic diversity in TAMs [25] and offers an avenue to explore cell–cell interactions within the glioma TME. However, our understanding of the heterogeneity and plasticity of TAMs during glioma progression remains limited. Several studies have highlighted the high heterogeneity of TAMs in grade 4 GBM, which extends beyond the linear M1/M2 transition paradigm [26, 27]. Grade 2–3 gliomas are known to be less aggressive than grade 4 GBM, apart from genetic differences among glioma grades, grade 2–3 is characterized by a less suppressive TME [28–30]. However, the analysis of the TME in grade 2–3 gliomas and grade 4 GBM alongside but separately is rarely conducted. Such an analytical approach may provide insights into glioma progression and assist in identifying key TAM subtypes for devising effective therapeutic strategies, including combined therapies.

Immunotherapies such as immune checkpoint blockade (ICB) and therapeutic vaccines have shown remarkable success in treating several types of cancer by harnessing the patient’s own immune response [31, 32]. However, their efficacy in inducing antitumor responses and achieving long-term remissions in glioma patients is limited. For instance, phase III clinical trials, such as CheckMate 498, CheckMate 548 and CheckMate 143, evaluated the use of Nivolumab (a programmed death 1 (PD-1) blockade antibody) alone or in combination with radiotherapy, temozolomide (TMZ), or both, failed to meet the primary endpoints in glioblastoma patients [33–35]. While some clinical trials have shown promise in GBM patients using personalized vaccines targeting tumor-specific antigens, not all patients benefit from this treatment [36, 37]. For example, the IDH1-vac, an IDH1 (R132H)-specific peptide vaccine, met its primary safety endpoint in a phase I trial involving 33 grade 3 and 4 *IDH1*(R132H)^+^ astrocytomas patients. Despite this, a subset of patients failed to mount a vaccine induced immune response and experienced disease progression [38]. Another allogeneic/autologous therapeutic vaccine that showed a survival benefit in a phase II study only achieved a 12-month survival rate of 40% [39]. Since the underlying causes of failed anti-tumor immune responses are not clear, it is essential to identify the barriers that hinder successful immune response in order to improve the effectiveness of immunotherapy for glioma treatment.

Genetic alterations in tumor cells can greatly influence the immune environment by regulating the recruitment, activation or suppression of immune cells. For example, Kirsten rat sarcoma virus (*KRAS*) mutations that are commonly found in colorectal and lung cancer, can induce immune evasion by producing immunosuppressive cytokines such as IL-10 and TGF-β [40]. *IDH* mutations can lead to the accumulation of 2-hydroxyglutarate (2-HG), which is known to suppress antitumor T cell immunity [41]. Nevertheless, the tumour-intrinsic alterations that dictate the TAM landscape in human gliomas are not well defined. A comprehensive understanding of cancer genomics and tumor immunology would reveal disease mechanisms and elucidate the limited success of targeted therapies such as epidermal growth factor receptor (EGFR) inhibition in glioma [42].

In this study, we performed an integrative analysis using both bulk and single-cell RNA-sequencing data to characterize the complex TME of glioma. Focusing on the heterogeneous TAMs, the current analyses identified three distinct TAM subsets, characterized by high expressions of either chemokine (C–C motif) ligand 3 (*CCL3*), allograft inflammatory factor 1 (*AIF1*), or secreted phosphoprotein 1 (*SPP1*). These subsets displayed differential associations with genetic alteration, T cell exhaustion and patient survival. These findings provide a rationale for developing new combination therapies. Moreover, a prognostic model was constructed based on the TAM landscape, which can predict anti-tumor immune responses and assist in risk assessment. These findings shed light on the heterogeneous immune environment underlying poor prognosis and provide targets for reprogramming the immunosuppressive TME for future treatment strategies.

## Results

### Analysis of cellular communication networks reveals tumor-promoting interactions between TAMs, tumor cells and lymphocytes

**Fig. 1:**
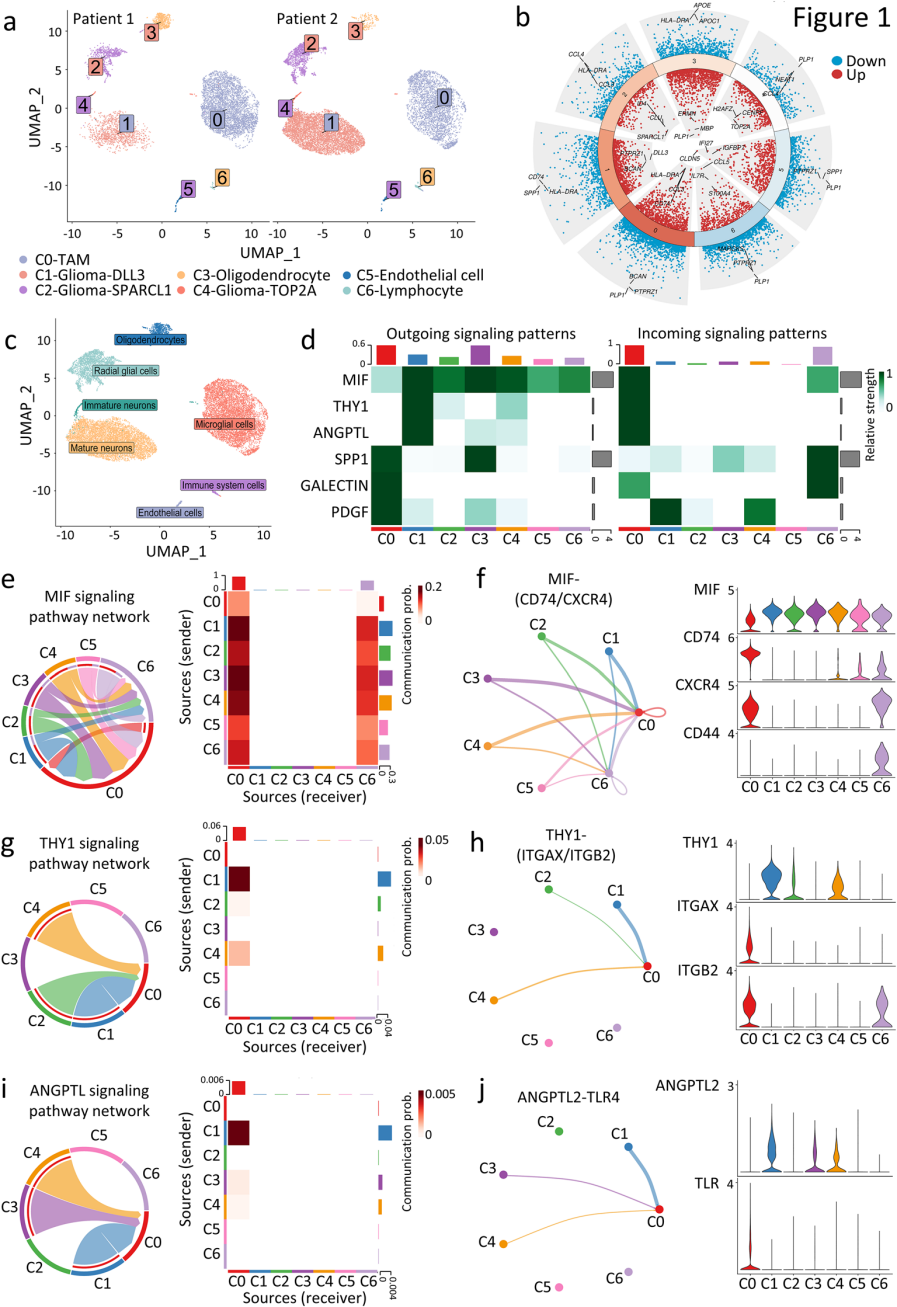
Illustration of cellular communication networks between tumor cells and TAMs in the low-grade glioma microenvironment. **a** scRNA-seq analysis of glioma patients. UMAP projection of 17,687 single cells isolated from tumor tissues, coloured by graph-based cell clusters and inferred cell types. **b** Volcano plot showing significantly upregulated or downregulated genes in each cluster, with the top three markers for each cluster highlighted. **c** UMAP projection of 17,687 single cells coloured according to automated cell type annotation by “ScType”. **d** Heatmap revealing the major MIF, THY1, ANGPTL, SPP1, GALECTIN and PDGF signals that significantly contribute to outgoing or incoming signaling for specific cell groups. **e**, **g**, **i** Chord plots and heatmaps showing significantly interacting pathways and communication probabilities of MIF (**e**), THY1 (**g**) and ANGPTL (**i**) pathways between tumor cells and TAMs in glioma TME. **f**, **h**, **j** Circle and violin plots depicting ligand-receptor pairs in each pathway and their respective expression patterns in each cell cluster

Considering that grade 4 GBM is characterized by a more suppressive TME than low-grade glioma [28, 29], we initially focused on examining the TME of two patients with low-grade glioma. To this end, we analyzed a publicly available single-cell RNA-sequencing dataset from four glioma tissues of two patients with low-grade glioma [26]. An initial quality control examination revealed 17,687 cells, with a median of 2,628 genes per cell. The data were analyzed using Uniform Manifold Approximation and Projection (UMAP) for dimensionality reduction and clustering. Seven distinct cell clusters were observed, namely Clusters C0 to C6 (Fig. 1a). Approximately 40% of the total cells were in Cluster C0, which highly expressed microglia markers *P2RY12* and *TMEM119*, along with macrophage markers *ITGAM* (encoding for CD11b), *CD68* and *CD14*. This population was designated as C0-TAM (Additional file 1: Fig. S1a, b). This is consistent with previous knowledge that TAMs represent a prominent immune cell population within the TME of gliomas [43]. By analyzing the expression of cell type-specific canonical markers (Fig. 1b), we categorized the remaining six clusters as follows: C1-glioma-DLL3, indicating gliomas with high cell surface expression of the notch ligand delta-like ligand 3 (*DLL3*) expression; C2-glioma-SPARCL1, referring to gliomas with elevated expression of acidic and rich in cysteine-like 1 (*SPARCL1*), which has been reported in cancer stem cell [44]; C3-oligodendrocyte; C4-glioma-TOP2A, characterized by high levels of topoisomerase II-alpha (*TOP2A*) expression, which is indicative of proliferating tumor cells [45], C5-endothelial cell and C6-lymphocyte (Fig. 1a). The above marker gene based manual annotation (Additional file 8: Table S1) demonstrated a high level of consistency with the automated cell-type scoring performed by ScType, a data-driven tool that enables cell type annotation through comprehensive cell marker databases (Fig. 1c and Additional file 8: Table S2) [46].

We then examined the intercellular communication between these seven cell clusters within the TME using the human CellChat database [47]. We analyzed the signaling patterns originating from and received by each of the seven cell clusters. Out of the 229 curated pathways, six signaling pathways, including migration inhibitory factor (MIF), thymus cell antigen 1 (THY1), angiopoietin-like protein (ANGPTL), osteopontin (SPP1), galectins (GALECTIN) and platelet-derived growth factor (PDGF) pathways were highly active in transmitting signals between different cell clusters (Fig. 1d).

The MIF signals sent by glioma cells (Clusters C1, C2 and C4) were mainly received by TAMs (Cluster C0) via receptors CD74 and CXCR4, and by lymphocytes (Cluster C6) via CD74, CXCR4 and CD44 (Fig. 1e, f). The MIF signaling axis on CD74/CXCR4 was found to be upregulated in glioma infiltrating macrophages and has been implicated in brain tumorigenesis by impeding microglial polarization [48, 49]. Meanwhile, ligand-receptor interactions between THY1 (CD90) and ITGAX/ITGB2 (CD11C/CD18) were observed from glioma cells (Clusters C1, C2, and C4) to TAMs (Cluster C0) (Fig. 1g, h). CD90 is a marker associated with mesenchymal stem cells in gliomas. Studies have reported that glioma tumor cells with high levels of CD90 are highly invasive [50, 51]. Additionally, CD11C/CD18 belongs to the family of integrins and is mainly expressed by myeloid cells. These adhesion molecules are known to potentiate cancer stem cell function [52, 53], supporting the role of the THY1-ITGAX/ITGB2 axis in glioma cell adhesion, survival, and differentiation. We also observed an enrichment of the ANGPTL signaling pathway between glioma (mainly from Clusters C1) and TAM (Cluster C0), mediated by angiopoietin-like 2 (ANGPTL2) and toll-like receptor 4 (TLR4) (Fig. 1i, j). On the other hand, TAMs signal the glioma cells through the PDGFB to PDGF receptor alpha (PDGFRα) axis (Additional file 1: Fig. S1c, d), which could trigger downstream phosphatidylinositol 3 kinase (PI3K) and mitogen-activated protein kinase (MAPK) pathways. This leads to tumor proliferation, metastasis, and angiogenesis in many malignancies [54]. The results suggest that TAM cells can interact with tumor cells in a reciprocal manner, facilitating glioma tumor growth.

To elucidate the mechanisms of TAM-mediated immunosuppression, we also investigated the interactions between TAMs and lymphocytes. Our findings revealed that the SPP1 and GALECTIN signaling pathways significantly contribute to the communication between TAMs (Cluster C0) and lymphocytes (Cluster C6) (Fig. 1d; 2a-d). These findings were in line with previous studies. For instance, the interaction between the glycoprotein SPP1 and the cell surface receptor CD44 has been found to inhibit T cell activation and proliferation, as well as promote tumor immune evasion [55, 56]. Additionally, Galectin-9 (encoded by *LGALS9*), a recognized suppressive immunomodulator, has been reported to confer immune tolerance by exhausting natural killer (NK) cells and stimulating regulatory T cell (Treg) differentiation through its interaction with CD44 [57, 58].

### Single-cell RNA-seq analysis reveals heterogeneous TAM subtypes in gliomas

**Fig. 2:**
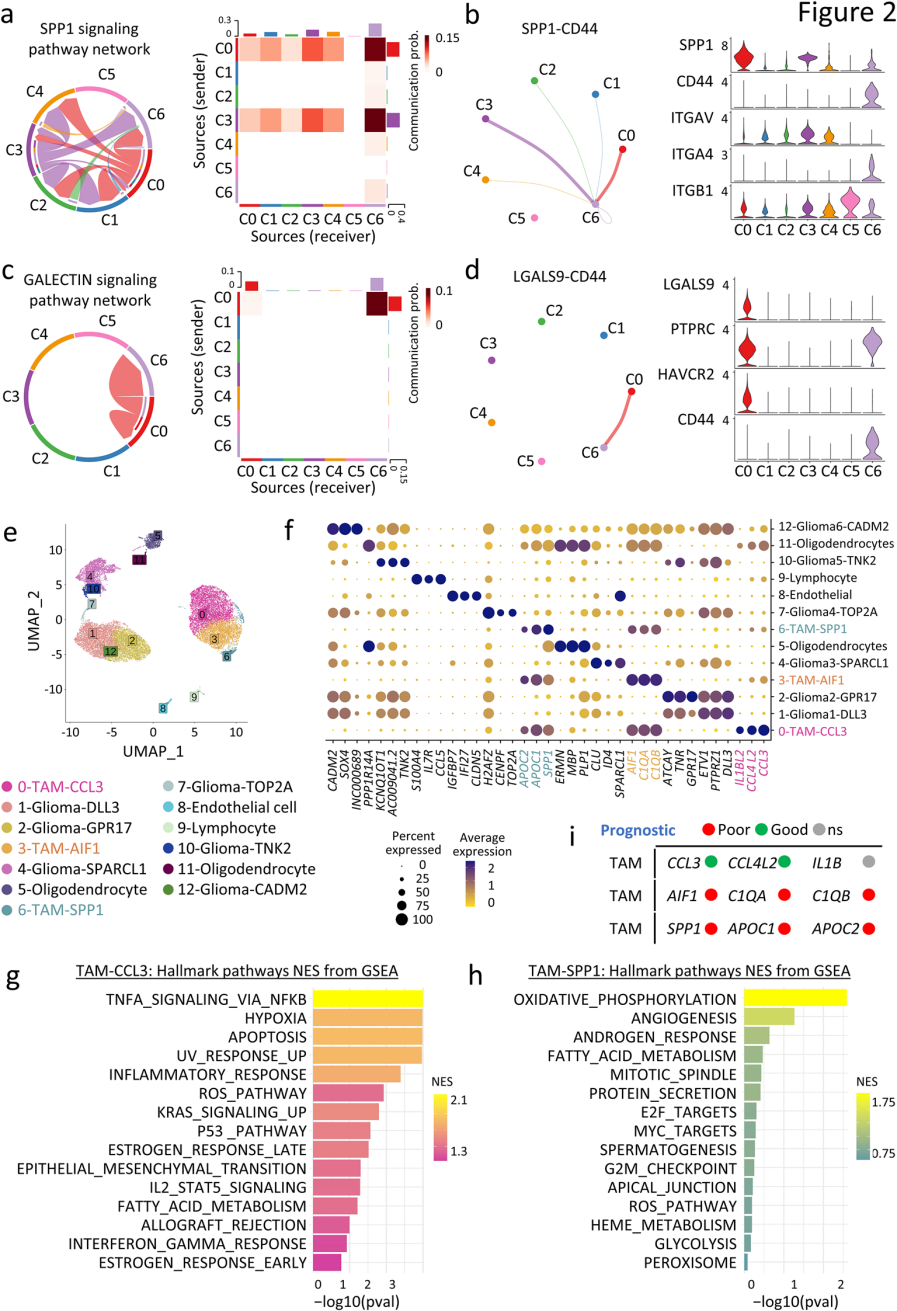
Illustration of cellular communication network between TAMs and lymphocytes as well as the characterization of TAM subsets in glioma patients. **a**, **c** Chord plots and heatmaps displaying significantly interacting pathways (SPP1 and GALECTIN) and communication probabilities between TAMs and lymphocytes in glioma TME. **b**, **d** Circle plots and violin plots indicating the ligand-receptor pairs in SPP1 and GALECTIN pathways and their expression patterns across cell clusters. **e** UMAP coloured by graph-based cell clusters and inferred cell types at an increased resolution. **f** Dot plot displaying three canonical markers among the top differentially expressed genes across clusters. **g**, **h** Hallmark pathway analysis showing the top 15 enriched categories of differentially expressed genes in TAM-CCL3 (**g**) and TAM-SPP1 (**h**) (Log_2_FC > 0.25, p < 0.05, n = 338 and 476) compared with other TAM subsets, coloured by normalized enrichment scores (NES) score. **i** Clinical prognostic prediction values for each TAM subtype in TCGA glioma patients. (with median value of gene expression used as cut-off. Green: high expression of biomarkers associated with better survival; red: high expression of biomarkers associated with poor survival ; ns: not significant)

To investigate the functional variations among TAMs in glioma, we characterized the TME at a higher resolution and further annotated it into 13 distinct cell clusters based on unique cell markers (Fig. 2e, f). Among these clusters, the TAM population (Cluster C0 in Fig. 1a) was further divided into three subsets and named as TAM-CCL3, TAM-AIF1, and TAM-SPP1 (Fig. 2e, f). The subset TAM-CCL3 exhibited relatively higher expression of chemokine-related genes, such as *CCL3, CCL4* and *CCL4L2,* which are known markers of active microglia (Fig. 2f and Additional file 2: Fig. S2a) [49, 59]. Pathway enrichment analysis conducted using ‘fgsea’, on the differentially expressed genes in the TAM-CCL3 subset compared to other TAM subsets, revealed an enrichment of immune activation pathways (p < 0.05). These pathways include tumor necrosis factor alpha (TNFα) signaling, inflammatory response, IL2 and signal transducer and activator of transcription 5 (STAT5) signaling, and interferon-gamma (IFNγ) response, suggesting its involvement in anti-tumor immune response (Fig. 2g). The TAM-AIF1 subset expressed high levels of *AIF1, C1QA, C1QB* and *P2RY12* (Fig. 2f and Additional file 2: Fig. S2a), which are markers of activated and homeostatic microglia [60, 61], suggesting that this population consist of brain-resident microglia. On the other hand, the TAM-SPP1 subset was characterized by high expression of *SPP1, FTL*, *LAPTM5*, *S100A11* (Additional file 8: Table S3). SPP1 is a secreted glycoprotein that highly expressed by bone marrow-derived monocytes and is known to sustain glioma cell survival and stimulate angiogenesis [62]. TAM-SPP1 subset also highly expressed lipid metabolism genes, including *APOC1, APOC2,* and *TREM2*, which are known markers of lipid-associated macrophages (LAMs) [63, 64] (Fig. 2e, f and Additional file 2: Fig. S2a). Pathway analysis indicated that TAM-SPP1 was enriched in angiogenesis, catabolic, and anabolic metabolic pathways (Fig. 2h). This implies that TAM-SPP1 utilizes the limited nutrients available in the TME by activating metabolic pathways, potentially playing a role in regulating metabolic hemostasis and angiogenesis within the glioma TME (Fig. 2h).

Next, we evaluated the clinical relevance of the three TAM subsets for prognosis prediction. Gene expression data from 507 low grade glioma patients were obtained from the TCGA database. We observed that patients expressing high levels of signature markers in the TAM-AIF1 and TAM-SPP1 subsets had poorer survival outcomes, whereas patients expressing high levels of markers in the TAM-CCL3 subset had better survival outcomes (Fig. 2i and Additional file 2: Fig. S2b). We therefore speculate that the composition of TAMs may significantly influence the prognosis of low-grade glioma patients.

### TAM subtypes exhibit distinct functions and are regulated by different transcriptional networks

**Fig. 3:**
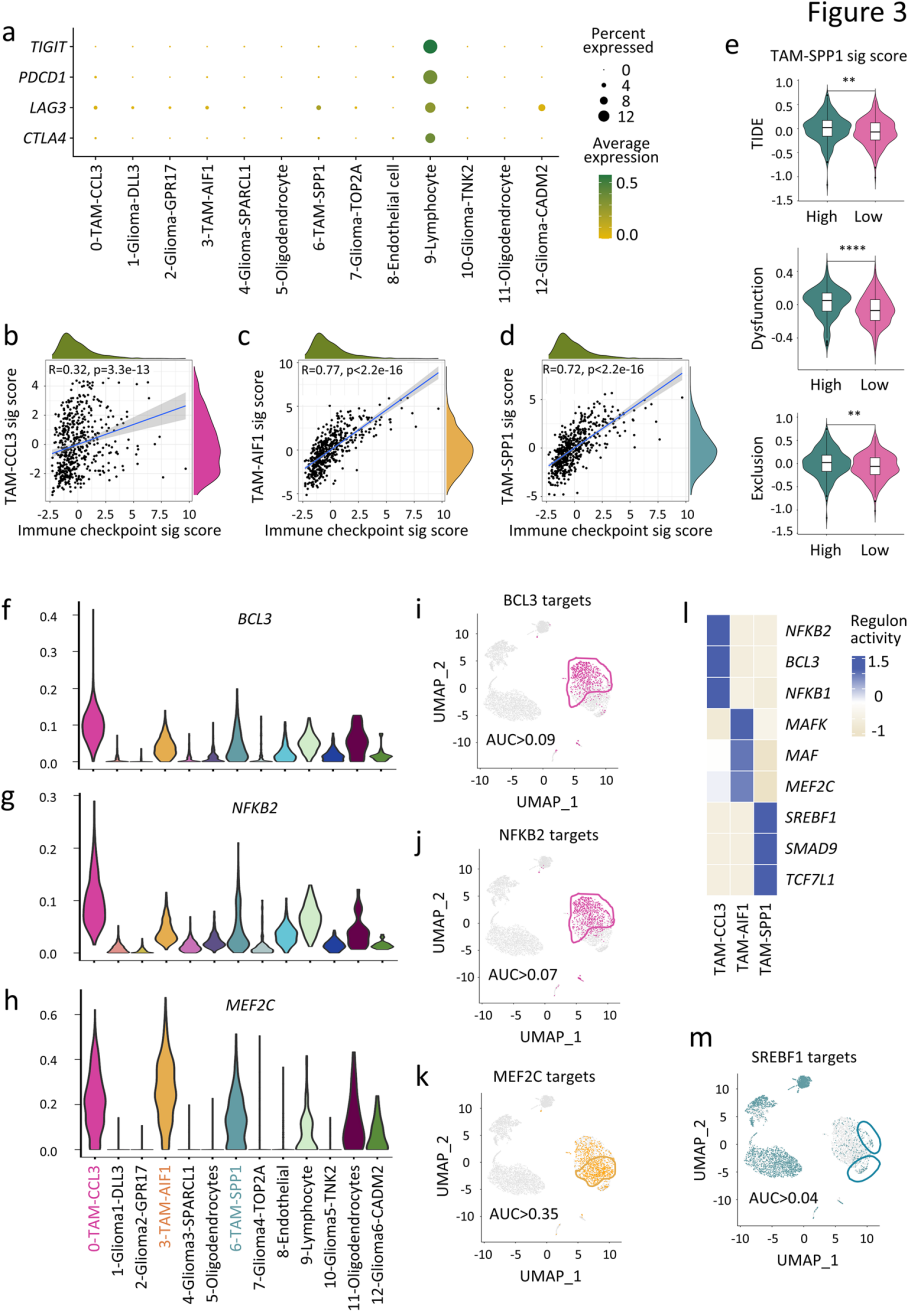
Characterization of distinct functions and diversely activated transcription factors within TAM subsets. **a** Dot plot indicating the expression of T cell exhaustion markers in each cluster of the glioma single cell dataset. **b**–**d** Spearman correlation between signature scores of the top five markers in TAM-AIF1 (*C1QB, C1QA, HLA-DRB1, AIF1, CD74*), TAM-CCL3 (*CCL3, CCL4, IL1B, CCL3L1, CCL4L2*), TAM-SPP1 (*SPP1, FTL, APOC1, S100A11, APOC2*), and the signature score of immune checkpoints (*CD274, PDCD1LG2, CTLA4, PDCD1, LAG3, TIGIT*) in the TCGA glioma dataset. **e** Analysis of TIDE, T cell dysfunction and T cell exclusion scores calculated by the TIDE algorithm in TCGA glioma patients with high and low marker scores for TAM-SPP1. **f**, **g**, **h** Violin plots showing expression of three TFs (*BCL3*, *NFKB2*, and *MEF2C*), selected as examples of enriched TFs in TAM-CCL3 or TAM-AIF1. **i**–**k**, **m** UMAP plots showing the regulon activity for TFs at the single-cell resolution, with cells having AUC scores higher than the threshold highlighted. **l** Heatmap showing top regulon activity in each TAM subtype. Statistical significance was determined by two-tailed Spearman correlation between variables for (**b**–**d**), and by unpaired two-tailed Student’s t-test for (**e**). **p < 0.01; ****p < 0.0001

We then investigated how TAM subsets exert their tumor suppression or promotion functions. Several immune-checkpoint molecules were substantially expressed in the lymphocyte cluster (Fig. 3a), including *CTLA4* (encoding Cytotoxic T-Lymphocyte Associated Protein 4, CTLA4), *LAG3* (encoding Lymphocyte-Activation Gene 3, LAG-3), *PDCD1* (encoding Programmed Death-1, PD-1) and *TIGIT* (encoding T-cell Immunoreceptor with Ig and ITIM domains, TIGIT) (Fig. 3a). Signature scores of immune checkpoints, including *CTLA4*, *LAG3*, *PDCD1*, *PD-L1*, *PD-L2* and *TIGIT* as well as the top five marker genes within each TAM subset were calculated using RNA-seq data from TCGA low grade glioma patients. Remarkably, the signature scores of immune checkpoints strongly correlated with the markers of TAM-AIF1 and TAM-SPP1 (p < 0.0001, R = 0.77 and 0.72, respectively) (Fig. 3b-d), suggesting that TAM-AIF1 and TAM-SPP1 might foster T-cell exhaustion in the TME. Moreover, Tumor Immune Dysfunction and Exclusion (TIDE) analysis, which integrates the expression signatures of T cell dysfunction and T cell exclusion to predict immunotherapy response [65], showed that the TAM-SPP1^high^ patients were significantly more susceptible to immunotherapy resistance based on higher TIDE (p < 0.01), T cell dysfunction (p < 0.0001) and T cell exclusion scores (p < 0.01) compared to TAM-SPP1^low^ patients (Fig. 3e). On the contrary, TAM-CCL3^high^ patients displayed lower TIDE (p < 0.05) and T cell exclusion scores (p < 0.01) compared to TAM-CCL3^low^ patients (Additional file 3: Fig. S3a). These results suggested that TAM-SPP1 plays the major role in suppressing T cells and could be a potential marker for predicting immunotherapy responses in glioma patients.

We hypothesized that different TAM subsets influence glioma progression through transcription factors (TFs) that regulate gene expression networks. Single-Cell Regulatory Network Inference and Clustering (SCENIC) was applied to infer TF-target gene interactions and define cell type-specific regulatory network activity [66]. The top three cluster-distinct TFs were identified for the 13 cell clusters (Additional file 3: Fig. S3b). The SOX TF family, which play an anticipated role in maintaining stemness and initiating the differentiation of stem cells in glioma [67], exhibited aberrant activity in some glioma cell clusters (Additional file 3: Fig. S3b). *SOX9* and *HEY2*, TFs associated with glioma stemness, were highly expressed in the Glioma-SPARCL1 population (Additional file 3: Fig. S3b), suggesting that this population might consist of glioma stem cells. In addition, *SOX10* and *NKX6.2* were highly expressed in oligodendrocytes, which have been reported to be involved in the differentiation and maturation of oligodendrocytes [68, 69]. *MECOM* and *EBF1* were highly active in endothelial cells, consistent with their known roles as endothelial lineage regulators [70, 71]. *BCL11B* and *PBX4* were highly expressed in lymphocytes (Additional file 3: Fig. S3b), consistent with their established roles in T cell development [72, 73].

The activity of the regulon (TF and its target genes) in each cell was qualified using the area under the curve (AUC) value, which reflects the proportion of regulon genes expressed in the cell. The regulon activity of the TAMs cell clusters provides a greater understanding of their functional heterogeneity. Notably, *BCL3* (encoding B-Cell Lymphoma 3) and *NFKB2* (encoding Nuclear Factor Kappa B Subunit 2), which serve as master regulators of inflammatory response, inflammasome activation, cytokine release, and cell survival [74], were enriched in the TAM-CCL3 subset (Fig. 3f, g). Cells with high BCL3 and NFKB2 regulon activity were shown on the UMAP plot, corresponded well with the TAM-CCL3 subset (Fig. 2, 3i, j). Moreover, myocyte-specific enhancer factor 2C (*MEF2C*), a marker of brain-resident microglia [75], was highly expressed in both TAM-AIF1 and TAM-CCL3 subsets (Fig. 3h) and showed higher regulon activity in these two subsets (Fig. 3k). The activation of lipid synthesis, as suggested by the high expression of SREBF1 regulon (Fig. 3l, m) as well as the enriched pathways of lipid metabolism and oxidative phosphorylation (OXPHOS) (Fig. 2h), indicated that TAM-SPP1 was likely undergoing metabolic reprogramming. Such changes suggest that TAM-SPP1 has a bias towards energy utilization, as enhanced OXPHOS would facilitate the anti-inflammatory function of macrophages [76, 77]. This single-cell analysis revealed the functional heterogenicity of TAMs within the glioma TME and uncovered the underlying transcriptional regulatory networks.

### Single-cell RNA-seq analysis reveals interaction between representative TAM subsets and exhausted T cells

**Fig. 4:**
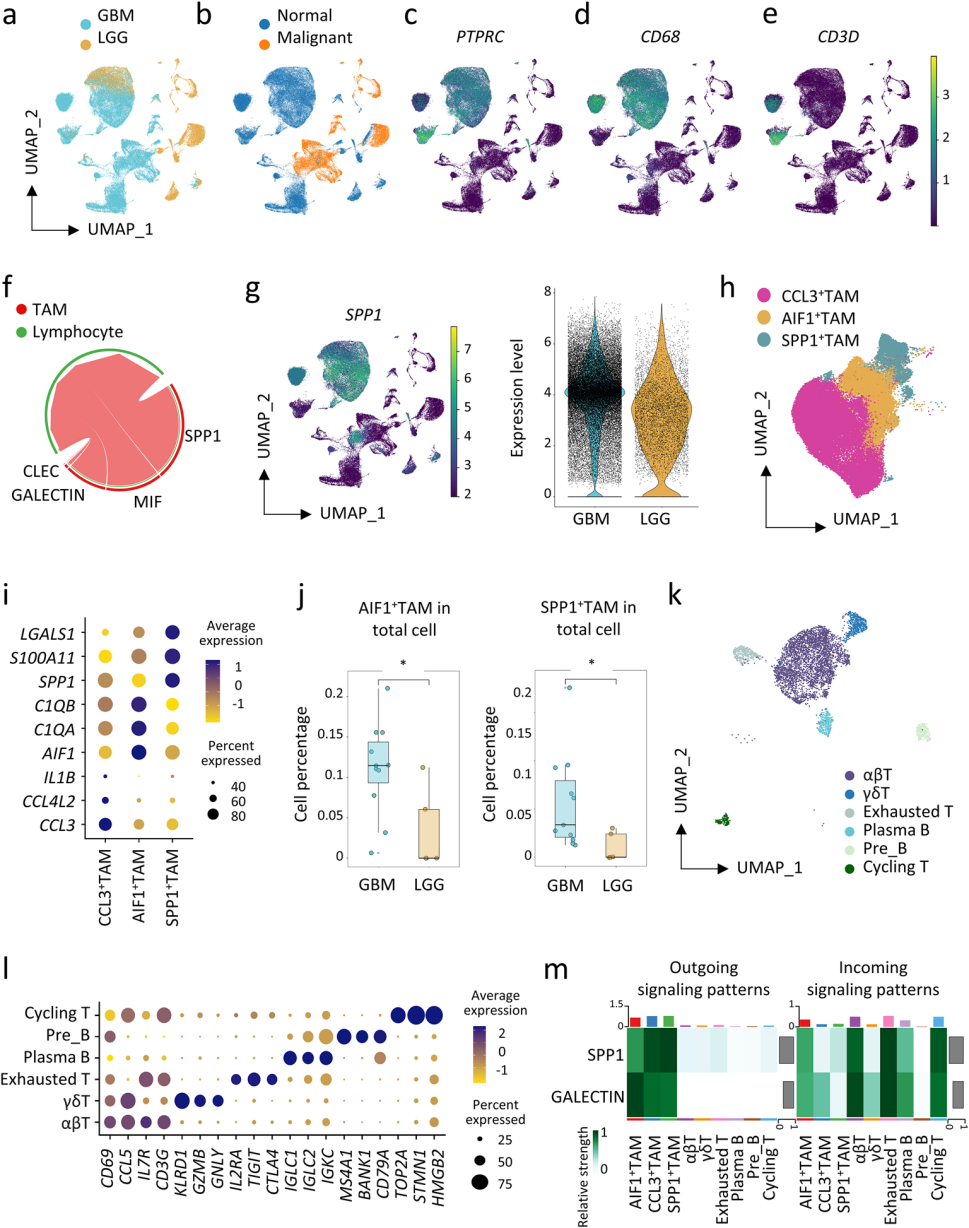
Single-cell RNA-seq analysis reveals interaction between representative TAM subsets and exhausted T cells. **a**, **b** UMAP projection of 122,626 single cells isolated from 17 glioma patients (six grade2/3 LGG and 11 grade 4 GBM samples), coloured by LGG/GBM (**a**) and normal/malignant cells (**b**). **c**–**e** UMAP plot coloured by expression pattern of *PTPRC* (**c**), *CD68* (**d**) and *CD3D* (**e**). **f** Chord plot showing the significantly interacting pathways between TAMs and lymphocytes in the glioma TME. **g** UMAP plot and violin plot showing *SPP1*’s expression pattern and levels in LGG and GBM. **h** UMAP projection of the three clusters of TAMs in the 17 glioma patients. **i** Dot plot indicating the expression of *CCL3*, *CCL4L2*, *IL1B*, *AIF1*, *C1QA*, *C1QB*, *SPP1*, *S100A11* and *LGALS1* in each cluster of the TAM subsets. **j** Box plot showing the proportion of AIF1^+^TAM and SPP1^+^TAM in total cells between LGG and GBM. **k** UMAP projection of six clusters of lymphocytes in the 17 glioma patients. **l** Dot plot indicating the expression of marker genes in each cluster of the lymphocytes. **m** Heatmap revealing the intensity of outgoing and incoming signals for TAM and lymphocyte subsets. Statistical significance was determined by unpaired two-tailed Student’s t-test for (**j**). *p < 0.05

This above analysis enabled us to investigate the TAMs in low-grade patients without the interference from aggressive high-grade glioma, which led to the identification of three distinct and easily distinguishable TAM populations. To ascertain whether such findings could be seen to larger datasets and to further elucidate whether these three distinct TAM subpopulations are associated with the aggressiveness of glioma, we extended the analysis across tumor grades by incorporating 122,626 cells from an additional 15 patients, including four grade 3 and eleven grade 4 glioma patients (Fig. 4a). Detection of large-scale copy number alterations by InferCNV analysis on the scRNA-seq data differentiating malignant cells from normal cells (Fig. 4b). Grade 4 GBM and grade 2–3 low grade glioma (LGG) have distinct transcriptomic profile for tumor cells, while they exhibited similar immune cell transcriptome profile (Fig. 4b, c). An examination of all significant cell–cell communications pathways between CD68^+^TAMs and lymphocytes (Fig. 4d, e) substantiated that SPP1 and GALECTIN signaling are predominantly responsible for the interactions between TAMs and lymphocytes (Fig. 4f). Notably, the TAM cells from grade 4 GBM expressed higher level of SPP1 compared to grade 2–3 LGG (Fig. 4g). We then performed clustering on the TAM subsets from the 17 cross-grade glioma patients and found three major TAM subsets with distinguished expression of *CCL3*, *AIF1*, and *SPP1* respectively, the three subsets also share most of the marker genes with the previously identified TAMs in grade 2 glioma (Fig. 4h, i and Additional file 8: Table S4). Interestingly, the AIF1^+^TAM and SPP1^+^TAM were significantly more prevalent in grade 4 GBM compared to grade 2/3 gliomas (Fig. 4j).

To better understand the lymphocyte subtypes, we performed de novo clustering on lymphocytes derived from 17 patients. This analysis revealed six major types of lymphocytes, including αβT cells, unconventional γδT cells, exhausted T cells, plasma B cells, pre-B cells and cycling T cells (Fig. 4k). Among these, the most abundant αβT cells was characterized by the expression of *CD3G*, *CD2*, *GZMK*, *CCL5* as well as *CD69*, the latter being a marker prevalent in tissue-resident rather than circulating T cells. Another exhausted T-cell subset exhibited elevated levels of immune checkpoint molecules and the activation marker CD25 (*IL2RA*) (Fig. 4l). Additionally, cell–cell interaction analysis between subsets of TAMs and T cells revealed that SPP1 signaling largely emanates from TAM-SPP1, whereas GALECTIN signaling primarily comes from TAM-AIF1 (Fig. 4m). We found that these exhausted T cells are one of the major recipients of both SPP1 and GALECTIN signaling, followed by αβT cells and cycling T cells (Fig. 4m).

Moreover, we examined a mouse dataset containing 12,228 single cells from non-tumor bearing mice, LGG, and high-grade gliomas (HGG) [49]. This dataset was generated using a spontaneous murine glioma model that recapitulates LGG-to-HGG malignant progression. Consistent with the findings in human gliomas, *Spp1*, *Aif1* and *Ccl3* mainly expressed in glioma-associated macrophages, hemostatic microglia and activated microglia, respectively (Additional file 3: Fig. S3c–e). Importantly, Spp1^+^TAMs were most abundant in HGG (n = 836) compared with LGG (n = 233) and less abundant in the non-tumor bearing mice (n = 112) (Additional file 3: Fig. S3f). Taken together, these results suggest a potential oncogenic role for SPP1^+^TAM in promoting glioma development. Moreover, monitoring the composition of TAMs, especially SPP1^+^TAM, in low-grade gliomas is important for prognosis prediction.

### TAM-SPP1 is associated with poor prognosis and worse survival in glioma patients

In the previous section, we highlighted the complex interplay between tumor and immune cells, and illustrated the heterogeneous functions of TAM subsets within the glioma TME. To corroborate these findings on a broader scale, we employed the deconvolution algorithm CIBERSORTx [78] to explore the composition of infiltrating immune cells in low grade glioma patients from the TCGA and the Chinese Glioma Genome Atlas (CGGA) datasets (Additional file 8: Table S5 and S6). Initially, we constructed a glioma-specific reference matrix based on the single-cell gene expression of the seven cell clusters mentioned earlier (Fig. 2e), which encompassed the three TAM subsets, as well as endothelial cells, glioma cells, oligodendrocytes, and lymphocytes. Subsequently, the customized gene-cell reference matrix was used to estimate the abundance of the seven cell clusters from the bulk RNA-seq datasets of TCGA and CGGA cohorts (Additional file 4: Fig. S4a). The bulk RNA-seq data were successfully deconvoluted with a median correlation of 0.817 (SD = 0.097) for the TCGA patients and 0.727 (SD = 0.182) for the CGGA patients.

**Fig. 5:**
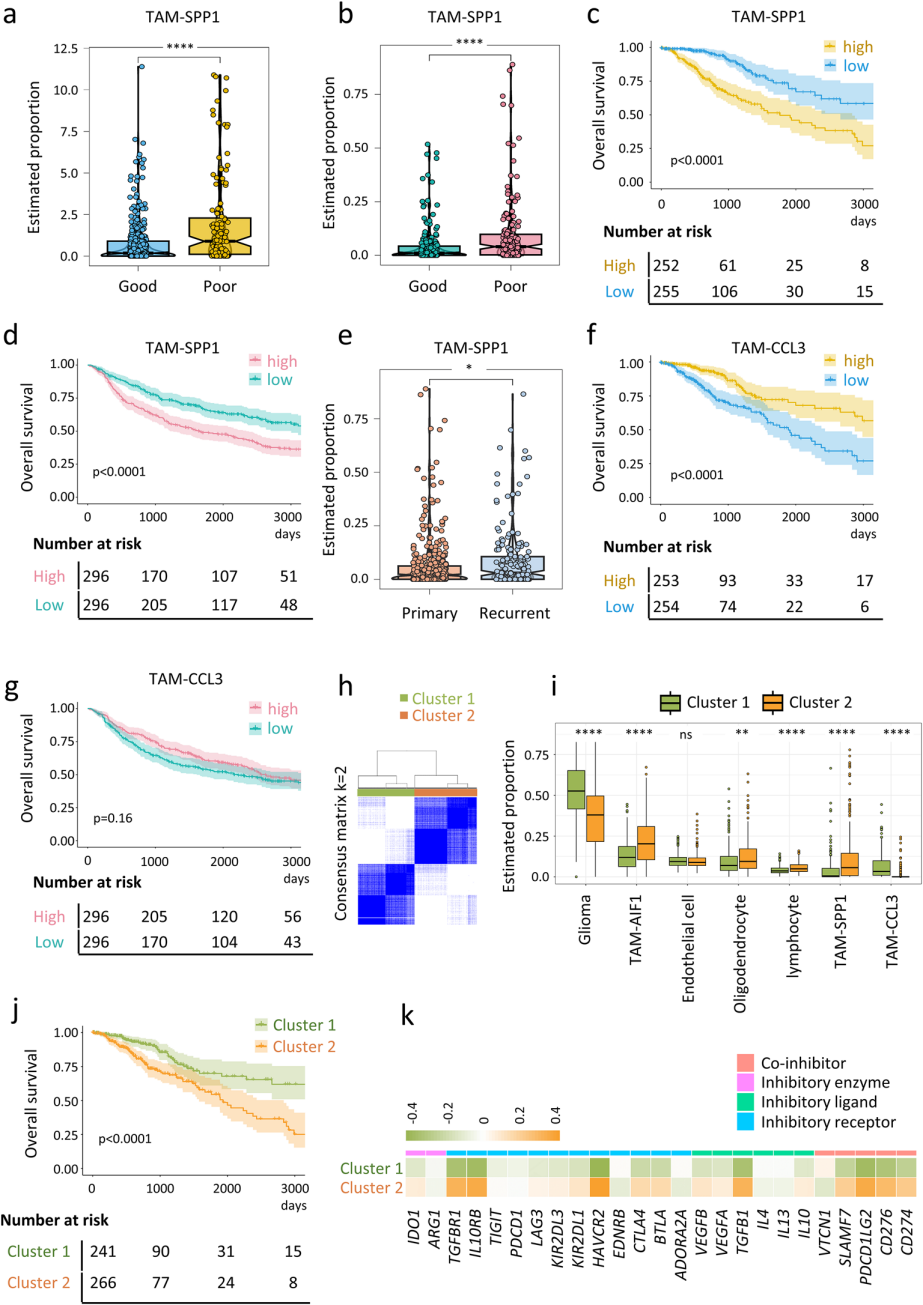
Survival analysis of TAM subsets deconvoluted from bulk RNA-seq datasets. **a**, **b** Boxplots showing the differential distribution of TAM-SPP1 in primary glioma patients from the TCGA (**a**) and CGGA (**b**) datasets (Good: alive; poor: death). **c**, **d** Kaplan–Meier curves of overall survival according to the proportions of TAM-SPP1 in TCGA (**c**) and CGGA (**d**) glioma patients. **e** Boxplots showing the differential distribution of TAM-SPP1 in primary and recurrent glioma patients from the CGGA dataset. **f**, **g** Kaplan–Meier curves of overall survival according to the proportions of TAM-CCL3 in TCGA (**f**) and CGGA (**g**) glioma patients. **h** Heatmap depicting a consensus clustering solution (K = 2) for three TAM signatures in 507 primary glioma samples from the TCGA dataset, the blue color indicating a high level of similarity in the expression profiles among the genes within a cluster. **i** Boxplot showing the differential distribution of seven cell types in Cluster 1 and Cluster 2 glioma patients from the TCGA datasets. **j** Kaplan–Meier curves of overall survival of patients in Cluster 1 and Cluster 2 among the 507 TCGA glioma patients. **k** Heatmap illustrating the expression pattern of inhibitory ligands, receptors, and enzymes in each cluster. Expression values are represented by z scores calculated across all tumors in the two clusters. (orange: high expression; green: low expression). Statistical significance was assessed by unpaired two-tailed Student’s t-test for (**a**, **b**, **e**, **i**), and by two-sided log-rank (Mantel-Cox) test for (**c**, **d**, **f**, **g**, **j**). *p < 0.05; **p < 0.01; ****p < 0.0001

Intriguingly, both TCGA and CGGA datasets indicated that TAM-SPP1 was consistently more abundant in primary glioma patients with poor prognosis (p < 0.0001, Fig. 5a, b), and was significantly associated with poorer overall survival compared to those with low levels (p < 0.0001, Fig. 5c, d). Recurrent glioma patients from the CGGA cohort also had elevated proportions of TAM-SPP1 compared to primary patients (Fig. 5e). Additionally, we noticed a trend towards better survival outcomes for patients with high levels of TAM-CCL3 (Fig. 5f, g). Higher level of TAM-AIF1 associated with poor overall survival of glioma patients in the CGGA cohort (Additional file 4: Fig. S4b). Overall, TAM-SPP1 emerged as one of the most profound predictors of glioma patient prognosis.

Next, we attempted to classify the TCGA glioma patients using the molecular signatures of the three distinct and easily distinguishable TAM populations. The patients were scored based on the expression of the top 15 markers within each TAM subset, segregating them into two distinct clusters, referred to as Cluster 1 and Cluster 2 (Fig. 5h and Additional file 8: Table S7). Cluster 2 patients showed elevated levels of TAM-SPP1 (p < 0.0001), TAM-AIF1 (p < 0.0001) and low levels of TAM-CCL3 (p < 0.0001, Fig. 5i), and experienced poorer survival outcome (Fig. 5j) compared to Cluster 1 patients. Consistent with the suppressor role of TAM-SPP1 in T cell inactivation through interacting with inhibitory checkpoints and secretion of suppressive cytokines [56, 79–81], we observed that Cluster 2 patients expressed higher levels of inhibitory immune molecules such as *CTLA4*, *LAG3*, *IL10,* and *TGFB1* (Fig. 5k), indicating a more suppressive TME. Considering the heterogeneity of TAMs, these results support our hypothesis that the composition of TAMs can serve as a prognostic predictor for glioma patients.

### A TAM signature-based prognostic model effectively predicts the anti-tumor immunity and accurately assesses the risk for glioma patients

Furthermore, we developed a prognostic model for glioma patients based on the molecular differences observed between patients in Cluster 1 and Cluster 2 (Additional file 5: Fig. S5a), who showed distinct prognosis, immune cell infiltration patterns and inhibitory molecules (Fig. 5i-k). Using a log_2_ fold change cut-off of 0.6, 1959 genes differentially expressed between Cluster 1 and Cluster 2 patients. Subsequently, a univariate Cox regression analysis detected 1311 genes significantly associated with patient outcomes. To further identify the most crucial variables associated with patient survival, a least absolute shrinkage and selection operator (LASSO) penalty Cox regression analysis was applied, and 17 genes were shortlisted. The 17 genes were ranked by the random forest algorithm (Additional file 5: Fig. S5b, c). Among the top 10 genes, four were found to be most predictive, including *APOBEC3C*, *EMP3*, *IGF2BP2* and *TGIF1* (Additional file 5: Fig. S5c). Using these four genes, a prognostic model was built for glioma patients through multivariate Cox regression analysis.

**Fig. 6:**
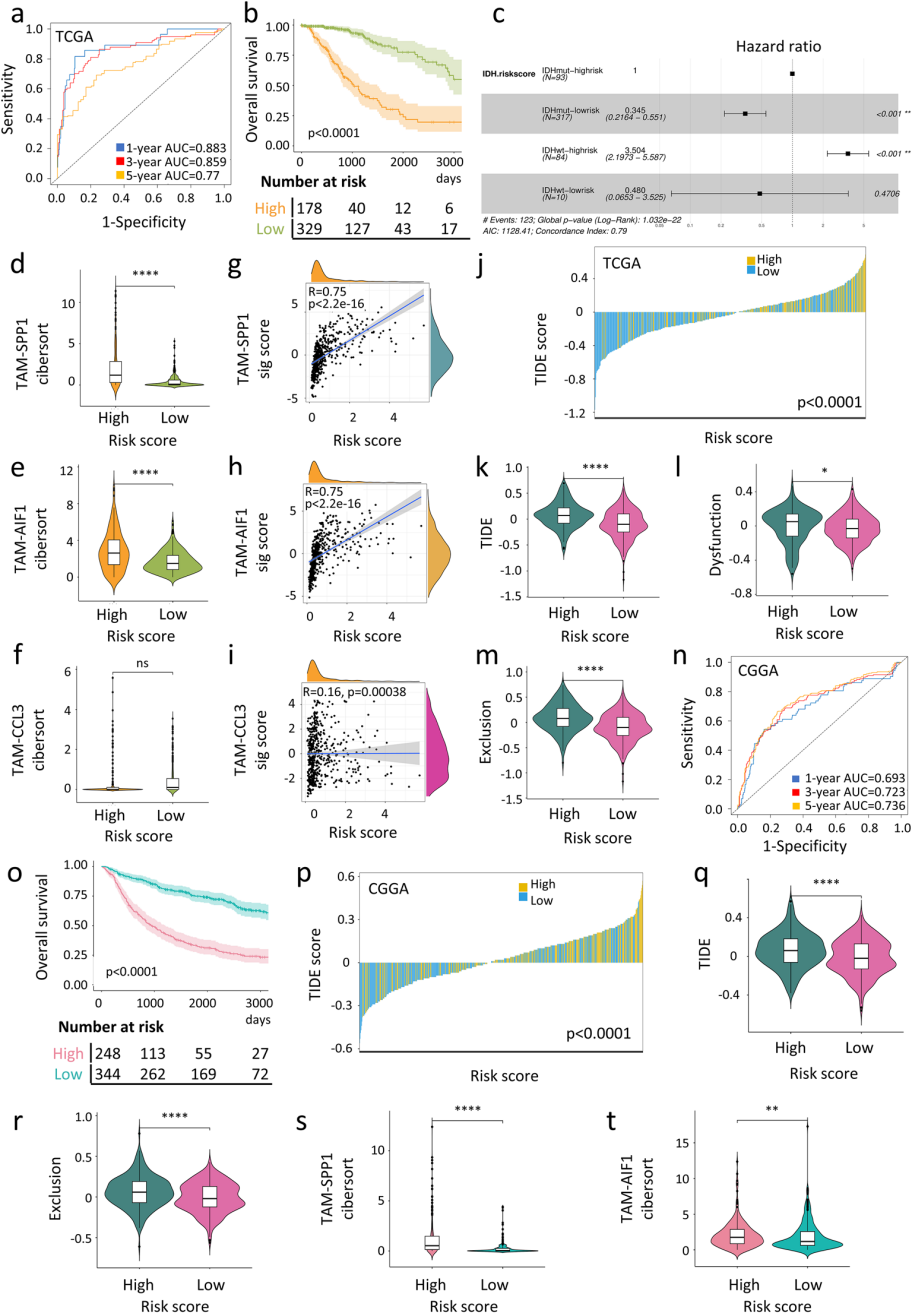
Assessment of prognostic performance and predictive power of the TAM signature-based model. **a** ROC curves of the prognostic model predicting 1/3/5-years survival in the TCGA glioma dataset. **b** Kaplan–Meier curves of overall survival in TCGA glioma dataset according to the high (n = 178) and low (n = 329) risk score calculated based on the prognostic model. The cut-off is selected using the point that maximizes the difference between the true positive rate (TPR) and the false positive rate (FPR). **c** Forest plot describing the associations between survival outcomes with risk scores from the prognostic model and *IDH* mutation. The p value was inferred by the univariate Cox regression model. **d**–**f** Violin plots representing the distribution of TAM-SPP1 (**d**), TAM-AIF1 (**e**) and TAM-CCL3 (**f)** between TCGA glioma patients with high (n = 178) or low (n = 329) risk scores. **g**–**i** Spearman correlation between risk scores and the signature score of the top five markers for each TAM cluster in the TCGA glioma dataset. **j** Prediction of potential clinical response to immunotherapy in TCGA glioma patients, comparing patients with high- versus low-risk score. **k**–**m** Analysis of TIDE (**k**), T cell dysfunction (**l**) and T cell exclusion (**m**) scores in TCGA glioma patients with high- and low- risk scores. **n** ROC curves of the prognostic model predicting 1/3/5-years survival in the CGGA glioma dataset. **o** Kaplan–Meier curves of overall survival in CGGA glioma dataset according to the high (n = 248) and low (n = 344) risk scores based on the prognostic model. The cut-off is selected using the point that maximizes the difference between the TPR and the FPR. **p** TIDE prediction of potential clinical immunotherapy response in CGGA glioma patients, comparing high- versus low-risk scores. **q**, **r** Analysis of TIDE (**q**) and T cell exclusion (**r**) scores in CGGA glioma patients with high and low risk scores. **s, t** Violin plots representing the TAM-SPP1 (**s**) and TAM-AIF1 (**t**) levels in CGGA glioma patients with high- and low-risk scores. Statistical significance was assessed by unpaired two-tailed Student’s t-test for (**d**–**f, k**–**m, q**–**t**), by two-sided χ^2^ test for (**j, p**), by two-tailed Spearman correlation between variables for (**g**–**i**) and by two-sided log-rank test for (**b, o**). *p < 0.05; **p < 0.01; ****p < 0.0001

The predictive performance of the model was then evaluated using time-dependent receiver operating characteristic (ROC) curves at 1, 3 and 5 years, which demonstrated an area under curve (AUC) exceeding 0.7 at each of these time points (Fig. 6a). Using the risk score derived from the prognostic model, patients were stratified into high- and low-risk groups, with the optimal cut-point in the ROC curve serving as the threshold. Kaplan–Meier analysis revealed a significantly poorer prognosis for patients in the high-risk group (p < 0.0001; Fig. 6b).

We assessed the prognostic ability of our four-gene prognostic model and established clinical risk factors using univariate Cox regression analyses. In addition to current well-known indicators of glioma prognosis, such as IDH mutation, 1p/19q co-deletion and MGMT promoter methylation, the risk score also represents a significant independent predictor for overall survival (Additional file 5: Fig. S5d). We then included all relevant clinical variables as cofactors in a multivariate Cox regression analysis. Importantly, the risk score remained the significant prognostic risk factor (HR 2.216, 95% CI 1.753–2.8, p < 0.001) (Additional file 5: Fig. S5e). This suggests that integrating the risk score with existing clinical indicators could enhance the overall survival predictions for glioma patients. Although patients with *IDH* mutation generally have better survival outcomes compared to patients with wildtype *IDH*, the survival rates still vary significantly. Therefore, we examined whether our developed prognostic model could provide a more accurate risk prediction based on the current risk assessment by *IDH* status. Univariate analysis results indicated that the risk score effectively further classified IDH-mutant patients into high- and low-risk groups (Fig. 6c). Consistent with the previous findings that patients in Cluster 2 had higher levels of TAM-SPP1 and TAM-AIF1 (Fig. 5i), patients with higher risk scores also demonstrated higher levels of TAM-SPP1 (p < 0.0001) and TAM-AIF1 (p < 0.0001) but not TAM-CCL3 (Fig. 6d-f). Moreover, the signature scores of marker genes in TAM-SPP1 (R = 0.75, p < 0.05) and TAM-AIF1 (R = 0.75, p < 0.05) displayed a stronger correlation with the risk score than TAM-CCL3 (R = 0.16) (Fig. 6g–i). These results indicate that the risk score can reflect the degree of immune suppression in the TME caused by different proportions of TAM composition.

In addition, high-risk patients showed significantly higher susceptibility to immunotherapy resistance (p < 0.0001; Fig. 6j) as indicated by their higher TIDE (p < 0.0001), T cell dysfunction (p < 0.05) and T cell exclusion scores (p < 0.0001) compared to low-risk patients (Fig. 6k, m), suggesting an immunologically barren TME in high-risk patients. This is in line with our earlier observations that TAM-SPP1 and TAM-AIF1 subsets were associated with T cell exhaustion (Fig. 3c, d).

Similar results were obtained when the prognostic model was evaluated using the CGGA dataset. The AUC values for 1-, 3-, and 5-year overall survival in the CGGA cohort were 0.693, 0.723, and 0.736, respectively (Fig. 6n). Patients with higher risk scores had poorer survival (p < 0.0001, Fig. 6o) and were predicted to have a poorer immunotherapy response (p < 0.001, Fig. 6p) based on higher TIDE and T cell exclusion scores (p < 0.0001, Fig. 6q, r). High-risk patients in the CGGA cohort also had higher levels of TAM-SPP1 (p < 0.0001) and TAM-AIF1 (p < 0.01, Fig. 6s, t). The risk score was also strongly correlated with the signature score of immune checkpoints (R = 0.74 and 0.72, p < 0.05, Additional file 5: Fig. S5f, g). Taken together, these results confirmed the robustness of the TAM-based prognostic model in indicating the functional status of T cells and predicting patient outcomes.

### *EGFR* amplification is associated with suppressive TAM subsets and a higher risk score

To investigate the genetic alterations associated with immunological differences and patient outcomes, we analyzed the somatic mutation profiles in the two clusters of glioma patients. According to single nucleotide variation (SNV) analysis, most variants in both clusters were missense variants, with Cluster 2 patients having approximately 15,000 SNPs and Cluster 1 patients less than 8000, predominantly cytosine to thymine substitutions (Additional file 6: Fig. S6a, b). The mutation types of the top 20 mutated genes were shown (Additional file 6: Fig. S6c, d). In both clusters, *IDH1*, *TP53*, *ATRX*, *CIC*, and *PIK3CA*, which are known to be involved in tumorigenesis, were frequently mutated genes. It is noteworthy that *EFGR* mutations were observed in 10% of patients in Cluster 2, whereas they were rarely seen in Cluster 1 patients (Additional file 6: Fig. S6c, d).

**Fig. 7:**
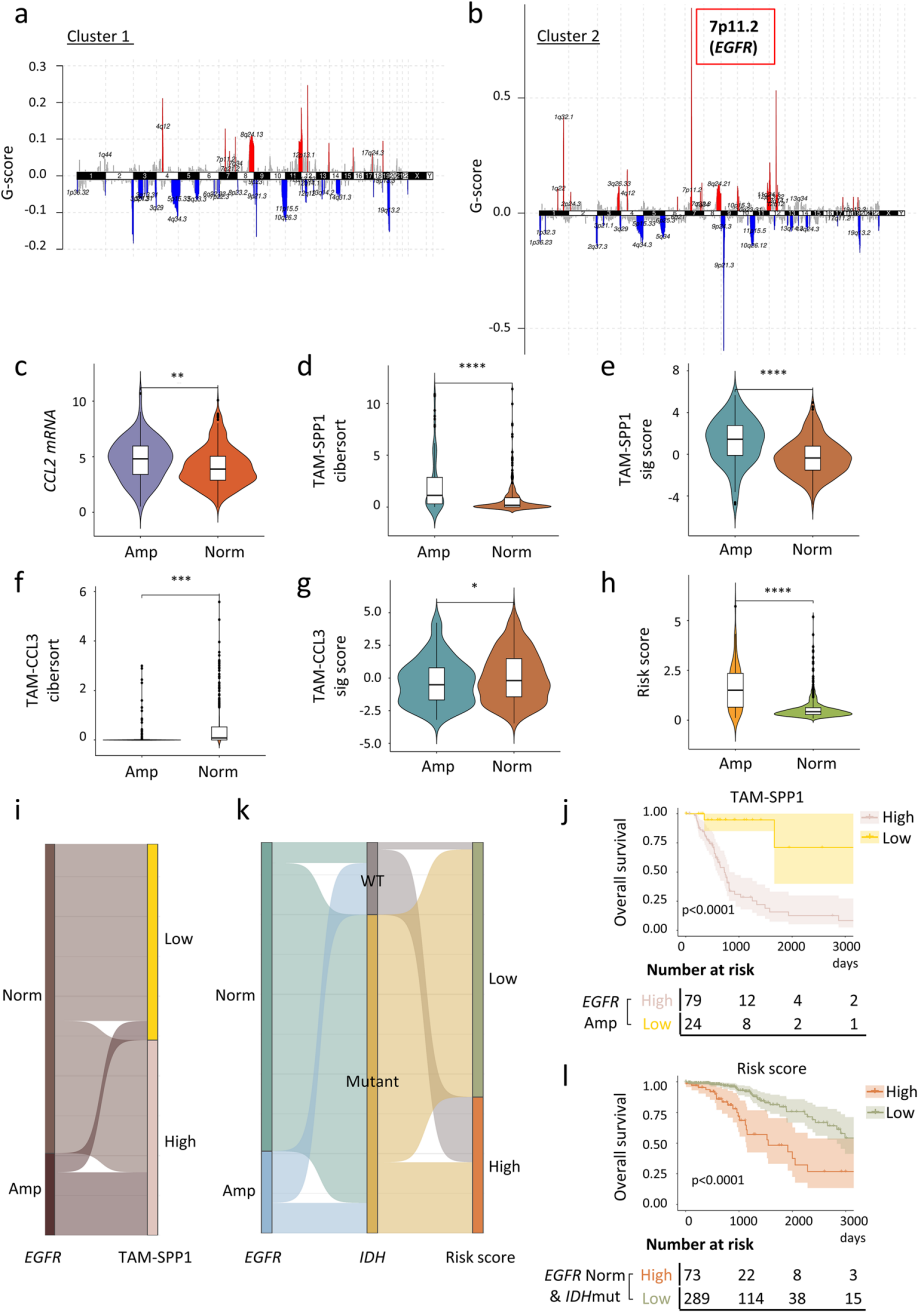
Association of *EGFR* amplification with TAM subtypes and risk score. **a**, **b** The distribution of GISTIC2.0 assigned G-scores for recurrent focal amplifications (red) and deletions (blue) in glioma Cluster 1 (**a**) and Cluster 2 (**b**) patients. **c** Violin plot showing the differential levels of *CCL2* mRNA [log_2_(TPM + 1)] expression between TCGA glioma patients with (n = 103) or without (n = 391) *EGFR* amplification. **d**, **f** Violin plots representing the differential levels of deconvoluted TAM-SPP1 (**d**) and TAM-CCL3 (**f**) proportions between TCGA glioma patients with (n = 103) or without (n = 391) *EGFR* amplification. **e**, **g** Violin plots showing the differential signature scores of marker genes for TAM-SPP1 (**e**) and TAM-CCL3 (**g**) between TCGA glioma patients with (n = 103) or without (n = 391) *EGFR* amplification. **h** Violin plot showing the differential levels of risk score between TCGA glioma patients with (n = 103) or without (n = 391) *EGFR* amplification. **i**, **k** Sankey plots visualizing the relationship between *EGFR* amplification, *IDH* mutation, TAM-SPP1 and risk score levels. **j** Kaplan-Meier curve of overall survival in *EGFR* amplified patients according to the high and low TAM-SPP1 levels. **l** Kaplan-Meier curve of overall survival in *EGFR* non-amplified and *IDH* mutant patients according to high- and low-risk scores calculated based on the prognostic model. Statistical significance was assessed by unpaired two-tailed Student’s t-test for (**c**–**h**) and by two-sided log-rank test for (**j, l**). *p < 0.05; **p < 0.01; ***p < 0.001; ****p < 0.0001

We also assessed somatic copy number variation (CNV) using GISTIC2, which calculates the G-score for each genomic location based on the frequency and magnitude of copy number changes [82]. For each cluster, a global CNV profile was obtained by selecting CNVs with a false discovery rate (FDR) q-value of less than 0.25. Interestingly, Cluster 2 patients showed a higher G-score for a 7p11.2 amplification which harbors *EGFR* (Fig. 7a, b and Additional file 6: Fig. S6e, f). This *EGFR* amplification was associated with higher *EGFR* mRNA expression and poorer survival outcomes (p < 0.0001, Additional file 7: Fig. S7a, b).

CCL2 plays a crucial role in recruitment of blood-derived monocyte and contributes to the immunosuppressive TME [83–85]. Targeting the CCL2/CCR2 axis has been shown to reduce tumor infiltrating macrophages in pre-clinical glioma studies [86, 87]. The single-cell data suggested that *EGFR* was primarily expressed in glioma cells (Additional file 7: Fig. S7c). Previous studies have shown that EGFR from tumor cells can regulate the recruitment of macrophages by increasing *CCL2* expression in glioblastoma and breast cancer [88–90]. Consistently, we observed higher levels of *CCL2* in patients with *EGFR* amplification (p < 0.01, Fig. 7c). Consequently, grade 2 and 3 glioma patients with *EGFR* amplification (copy number fold change > 1.2, n = 103) had higher level of TAM-SPP1 (p < 0.0001) as well as higher score of TAM-SPP1 marker genes compared to patients without *EGFR* amplification (Fig. 7d, e). On the other hand, patients with amplified *EGFR* showed lower level of TAM-CCL3 (p < 0.001) and lower score of TAM-CCL3 marker genes (Fig. 7f, g). This observation was consistent with previous findings (Fig. 5i), implicating that *EGFR* amplification may serve as one of the regulators in depicting the TAMs landscape. In support of our hypothesis, most of the *EGFR*-amplified patients had high levels of TAM-SPP1 (Fig. 7i). Interestingly, these patients showed poorer overall survival compared to *EGFR*-amplified patients with low levels of TAM-SPP1 (p < 0.0001, Fig. 7j), indicating that the recruitment of TAM-SPP1 may underscore the oncogenic role of *EGFR* amplification. This finding is corroborated by a well-annotated single-cell dataset encompassing 92,102 myeloid cells from 62 patients with grade 4 GBM [91]. Among these cells, 35,391 are from patients with wild type *EGFR*, while the remaining cells are from patients with amplified *EGFR*. We leveraged the systematic annotation provided by the GBM atlas. The results showed that two groups of bone marrow derived TAMs (TAM-BDM) (namely TAM-BDM anti-inflammatory and TAM-BDM hypoxia/mesenchymal (MES)) were more prevalent in patients with amplified *EGFR* compared to those with wild type *EGFR*. Notably, these two groups of TAMs showed high levels of *SPP1* but low levels of inflammatory cytokines such as *CCL4* (Additional file 7: Fig. S7d, e).

In line with this, patients with *EGFR* amplification had significantly higher risk scores (Fig. 7h) and stronger expressions of the four prognostic markers utilized in the construction of the risk score model (Additional file 7: Fig. S7f–i), compared to patients without *EGFR* amplification. This is consistent with the role of the prognostic model in depicting the degree of immune suppression that arises from changes in the composition of TAMs. On the other hand, most *EGFR* non-amplified patients harbored an *IDH* mutation (Fig. 7k), and they could be further stratified into low- and high-risk groups (Fig. 7l) based on the prognostic model. Thus, this model may provide additional risk management information for glioma patients with *IDH* mutations. Taken together, our findings highlight the association between *EGFR* amplification and TAM-SPP1 and provide novel approaches that complement existing risk stratification methods.

## Discussion

In the current study, we aimed to identify factors that contribute to impaired immunosurveillance and the variation in outcomes for grade 2 and 3 gliomas, which have not been fully elucidated. By single-cell analyses, we revealed a comprehensive interaction network within the glioma TME, with TAMs playing pivotal roles in tumor progression by interacting with both tumor and T cells. Corroborating evidence from both bulk and single-cell RNA-seq analyses highlighted the diversity of TAMs, characterized by heterogeneous phenotypes and distinct regulatory networks. Among the three TAM subsets, TAM-SPP1 was notably associated with diminished T cell response and poorer patient outcomes. These analyses shed light on how to augment anti-tumor immune response and revitalize immunosurveillance by manipulating TAM-SPP1 levels or impeding TAM-cell interactions. These findings would provide rationale for combination strategies to mitigate tumor cell activity and prevent disease progression. Importantly, a four-gene prognostic model constructed based on the TAM molecular pattern, has demonstrated superior predictive ability for predicting survival outcomes in both TCGA and CGGA cohorts. It also shows improved risk prediction when combined with current predictors that primarily focus on genetic alterations. In addition, the risk score calculated based on the model can be used to predict immune response to immunotherapy. Additionally, we discovered a significant association between *EGFR* amplification and both the level of TAM-SPP1 and the predicted risk score, implicating a potential link between genetic changes and immune remodeling. Collectively, the results underscore the pivotal role of TAMs in the suppressive TME and suggest potential therapeutic targets to overcome immune suppression in glioma. These findings also guide the development of more refined clinical markers for better patient risk stratification, aligning with current standards.

Accumulating evidence suggests that microenvironment remodeling may have a more pivotal role in glioma evolution than tumor-intrinsic changes [92, 93]. Therefore, apart from targeting tumor-intrinsic factors, therapeutic interventions overcome extrinsic hurdles are crucial for eliminating therapy-resistant residual cells, improving treatment response and patient outcomes. The strategies for eliminating resistant cells and improving patient outcomes in grade 2 and 3 gliomas remain to be established. Our analysis identifies critical ligand-receptor pairs between TAMs and glioma cells that promote tumor progression, such as MIF-CD74/CXCR4, THY1-ITGAX/ITGB2, and ANGPTL2-TLR4. Therefore, blocking these ligand-receptor interactions may serve as potential therapeutic approaches for clinical management. For instance, targeting MIF signaling could sensitize chemotherapy [94], as MIF has been found to cause temozolomide resistance by activating the tumor intrinsic PI3K/AKT signaling pathway [95]. Also, a recent report suggested that THY1-positive tumor cells colocalized with TAMs in GBM, and this was associated with recurrence [96]. Interestingly, we also observed an increased abundance of TAM-SPP1 in recurrent glioma patients and their interaction with tumor cells via THY1-ITGAX/ITGB2 signaling. Lastly, our findings support the idea that gliomas may promote the accumulation of suppressive macrophages in the TME through the ANGPTL2-TLR4 interaction, as ANGPTL2 has been implicated in macrophage accumulation in adipose tissue and polarization towards M2 macrophage in other types of cancer [97, 98].

In addition to the interactions between tumor cells and TAMs, the current analysis also illustrates the suppressive effect of TAMs on T cell activity through SPP1-CD44 and LGALS9-CD44 interactions, which mainly originate from TAM-SPP1 and TAM-AIF1 and received by exhausted T cells. These findings are supported by a previous study on colon carcinoma, which indicated that SPP1 could dampen T cell activation and confer tumor immune tolerance through binding to CD44 [56]. Similarly, the LGALS9-CD44 interaction has been shown to enforce differentiation and maintenance of suppressive regulatory T cells [57]. By using CD44-specific blocking antibodies or aptamer [99], it may be possible to reverse the immunosuppression on gliomas through the blockage of CD44 signaling. Taken together, our work offers a rationale for combining therapies that block these interactions with conventional chemoradiotherapy to eradicate resistant tumor cells in grade 2 and 3 glioma.

In this study, we identified three TAM subsets in gliomas (Fig. 2e, f, h). Among these subsets, TAM-CCL3, representing activated microglia, exhibits elevated levels of cytokines and chemokines, such as *CCL3* and *CCL4*. These marker genes have been identified in inflammatory macrophages in other types of cancer such as spinal ependymomas and osteosarcoma [100–102]. They play a crucial role in inflammatory response through recruiting NK cells and T cells within the TME [103, 104]. On the other hand, TAM-AIF1, characterized by the high expression of *C1QB, C1QA, HLA-DRB1*, is likely a type of glioma-specific tissue-resident microglia suggested by their high expression of microglia markers such as *P2RY12*, *TMEM119*. In support of this, a group of tissue resident macrophage (C1Q^+^macrophages) has been reported to express similar marker genes (*C1q*, *HLA-DR*) in several other tumor types. These cells exhibited higher M2 signatures and have been associated with T cell exhaustion and tumor progression [105, 106]. This indicates that these tissue-resident macrophages transition to an immunosuppressive phenotype in the TME. Zhang et al. utilized in vitro co-culture assays to demonstrate that C1q^+^TAMs impair the antitumor responses of both mouse and human CD8^+^T cells [107]. Moreover, TAM-SPP1, representing bone marrow-derived macrophages, is characterized by high expression of lipid-related genes like *APOC1*, *APOE*, and *TREM2*. Existing evidence suggests that SPP1^+^TAM and TREM2^+^TAM (also known as lipid-associated macrophages) are abundant in various types of cancers, such as colon cancer [108], lung cancer [63], and hepatocellular carcinoma [109, 110]. These macrophages exhibit elevated M2 signatures and are often associated with inferior outcomes [106]. These macrophages are recognized as key mediators of immunosuppression through the secretion of suppressive cytokines [80], upregulation of checkpoint expression [110] and enhancement of interactions with T cells [79]. Despite evidence showing SPP1^+^TAM promoting glioma cell survival by secreting SPP1 or interacting with tumor cells [62, 111, 112], it is unclear how this population is regulated or how it affects T cell function in gliomas.

The current study elucidated the regulatory network and functional role of TAM-SPP1 in grade 2 and 3 gliomas (Fig. 2e, f, h). We found that TAM-SPP1 was metabolically reprogramed towards lipid metabolism and oxidative phosphorylation, potentially driven by the SREBF1 transcription factor. In addition, TAM-SPP1 is strongly associated with T-cell dysfunction and exclusion, as well as poorer patient outcomes, indicating that this population could serve as a therapeutic target. This hypothesis is supported by previous studies on multiple cancers, which have proposed therapies that target SPP1 or disrupt the interaction between SPP1^+^TAM and other cells as potential treatment for multiple types of cancer. For example, targeting the interaction between SPP1^+^macrophages and fibroblasts can enhance immunotherapy response in colorectal cancer [113]. Moreover, sensitivity to cisplatin was also improved in a mouse ovarian tumor model using anti-SPP1 and anti-CD44 antibodies [114]. Additionally, blockade of SPP1 led to enhanced efficacy of immunotherapy in hepatocellular carcinoma [115]. The anti-osteopontin monoclonal antibody AOM1 (Pfizer Inc) was found to significantly inhibit lung tumor growth [116]. On the other hand, depletion of TREM2^+^LAM suppressed tumor growth in breast cancer mouse model [117] and improved anti-tumor immunity and immunotherapy efficacy [118, 119]. A recent study suggested that TREM2 inhibition remodels macrophages into an immune-active functional status, increases anti-PD-1 efficacy and inhibits tumor growth in a glioblastoma mouse model [120]. The current analyses indicate that targeting SPP1 or TREM2 can inhibit immunosuppressive TAMs, thus representing potential therapeutic targets. With the emergence of monoclonal antibody targeting TREM2 [119], this avenue of treatment seems promising for modulating the immune environment in glioma patients.

Histological and genetic anomalies are currently considered the standard for diagnosing and stratifying the risk of glioma patients. Despite the importance of TAMs in glioma progression, there is a lack of risk models that incorporate composition of TAMs. In this study, we not only unravelled the TAM landscape and dissected TAM functional heterogeneity in glioma, but also constructed a prognostic model based on differentially expressed genes found in two glioma molecular subtypes with disparate TAMs-related signatures and clinical outcomes. Methods including univariate Cox, LASSO regression, and random forest were used to develop the model, which successfully controlled confounding variables. The model encompasses four crucial genes (*APOBEC3C*, *EMP3*, *IGF2BP2*, *TGIF1*) and demonstrates robust capability in predicting patient outcomes, as evidenced by its strong prognostic accuracy in two independent cohorts. Importantly, our model offers an enhanced level of prognostic accuracy particularly for patients harboring the IDH mutation, who are typically considered to have a favorable prognosis. This refinement enables the identification of individuals who may require closer observation due to an elevated risk of adverse prognosis. Furthermore, the risk score calculated based on the model correlates strongly with high levels of TAM-SPP1, T cell dysfunction, exclusion, and impaired T cell response. This indicates that the model can be applied to predict anti-tumor immune responses. Taken together, with the prognostic model’s capability of predicting T cell functional status, it could potentially serve as a useful tool for predicting both patient risk and immunotherapy response.

Specific genomic alterations such as amplification and activating mutations in the *EGFR* have been identified as one of the causes of initiating glioma [121]. However, the mechanisms by which genetic alterations influence the glioma TME remain largely unexplored. Beyond EGFR’s classical role in promoting glioma cell proliferation and survival through the activation of oncogenic PI3K, AKT and RAS/MAPK pathways, EGFR overexpression has been linked with macrophages recruitment in GBM and breast cancer [88, 89]. Moreover, *EGFR* has been associated with high TREM2^+^TAM infiltration, advanced tumor progression and inferior prognosis in lung cancer patients [63]. EGFR-targeted therapy has been shown to reduce M2 macrophage levels by diminishing chemokine expression [88, 89]. On the other hand, TAMs can secrete EGF, which stimulates EGFR in glioblastoma cells, thereby reinforcing the oncogenic circuit [122]. Here we demonstrated that patients with *EGFR* amplification predominantly exhibit elevated levels of TAM-SPP1. Importantly, we observed that *EGFR*-amplified patients with lower levels of TAM-SPP1 show significantly better survival compared to those with high levels of TAM-SPP1. This indicates that targeting TAM-SPP1 may help reverse disease progression in *EGFR*-amplified patients. Although *IDH* mutation is considered as a marker of longer overall survival time and better therapeutic response [123], patient prognosis varies widely. Our prognostic model allows for a more precise risk assessment, particularly for patients harboring *IDH* mutations and lacking *EGFR* amplification.

The current study provides prognostic value by constructing a risk-prediction model and reveals potential targets for combined therapies. Nonetheless, several limitations were acknowledged in this study. First, the identification of cell type from single-cell analysis relied on publicly available resources with limited number of patients. It limits the capability of detecting rare cell subtypes and dissecting highly heterogeneous cell clusters, or depicting the heterogeneity and dynamic differentiation status of tumor-infiltrating T cells. In the future, studies with larger datasets will be beneficial to gain a better understanding of disease-related rare cell subtypes and their associations with clinical phenotypes. The development of more robust models could be achieved by focusing on more precisely defined immune cell types. Second, the current analyses primarily focused on data mining and were validated by existing literature. Additional studies may be necessary to determine the functional utility of the identified pathways in targeted treatment in the future. Third, the current study observed the association between *EGFR* amplification, *CCL2* upregulation and accumulation of TAM subtypes, which is supported by studies in GBM and breast cancer. However, future in vitro or in vivo studies are necessary to fully understand their mechanistic linkage.

## Methods

### Preprocessing, dimension reduction and clustering of scRNA-seq data

The human glioma scRNA-seq datasets [26, 91, 124] were downloaded from GEO (GSE182109, GSE200984) and cellxgene (https://cellxgene.cziscience.com). The mouse glioma scRNA-seq datasets were downloaded from GEO (GSE221440) [49]. All datasets used in this study were summarized in Additional file 8: Table S8. For the reanalysis of human glioma datasets, cells with less than 500 or more than 20,000 UMIs, over 20% mitochondrial genes and more than 0.1% hemoglobin genes were filtered out. “Seurat” (Version 4.3.0) was applied for data normalization, finding variable genes, scaling (NormalizeData, FindVariableFeatures and ScaleData functions), principal component analysis (RunPCA), dimension reduction (RunUMAP) and unsupervised graph-based clustering (FindNeighbors & FindClusters). “Harmony” (Version 0.1) was used to remove batch effect (RunHarmony) [125]. Differentially expressed genes (DEGs) for each cluster were identified (FindAllMarkers) and visualized via UMAP plots, heatmaps, violin plots and volcano plots. “scRNAtoolVis” (Version 0.0.4) was used to plot markers in each cluster (jjVolcano) with an absolute cutoff value of 0.5 for log2 fold change and a threshold of 0.01 for adjusted p-value. DEGs between the specified clusters were identified (FindMarkers). Gene set enrichment analysis of DEGs with an adjusted p-value less than 0.05 was conducted for each cluster using the fgsea package (Version 1.24.0) [126]. The analysis utilized the MSigDB hallmark gene sets (h.all.v2023.1.Hs.symbols.gmt) to identify significant enrichment patterns. The automated cell type annotation was conducted using ScType (Version 1.0) [46], targeting “Brain” as the tissue of interest. The inference of large-scale copy number alterations was performed using inferCNVpy, tumor cells were defined by CNV score over 0.01 (Version 0.4.3).

### Cell–cell communication network construction

“CellChat” (Version 1.5.0) [47] was applied to construct and visualize intercellular communication networks. CellChat database for humans was used which includes 1,939 validated interactions. Downstream analysis included inferring cell–cell communication probability (computeCommunProb and filterCommunication), and communication probability at both signaling pathway and gene levels (computeCommunProbPathway). Significant signaling pathways and ligand-receptor pairs were visualized as circle plots (netVisual_individual), heatmaps (netVisual_heatmap, netAnalysis_signalingRole_heatmap) and chord plots (netVisual_aggregate, netVisual_chord_gene).

### Single-cell regulatory network inference

“SCENIC” (Version 0.12.1) was used for the inference on TF regulatory networks at the single-cell level [66]. The expression data of TAMs were used as input and potential TFs were identified (GENIE3). Known human TF motif databases annotated at 500 bp and 10 kb of transcriptional start sites were used for TF-motif enrichment and TF’s direct targets identification (CisTarget). The activity of regulons on single cells were scored (AUCell). The AUC thresholds were computed (get_regulon_thresholds). Cell type-specific TFs and regulons were visualized using heatmaps, violin plots and UMAP.

### Processing bulk RNA-seq data

The clinical and RNA-seq data of low-grade glioma patients from The Cancer Genome Atlas (TCGA) and the Chinese Glioma Genome Atlas (CGGA) were downloaded from the NCI Genomic Data Commons (GDC) repository and http://www.cgga.org.cn/, respectively. For each patient, the signature scores of each TAM cluster’s marker genes and immune checkpoints were calculated based on the transcripts per million (TPM) of gene expression using the calculate_sig_score function of IOBR package with parameter “method = PCA” [127]. TIDE, T cell dysfunction and T cell exclusion scores were calculated using the TIDE algorithm (http://tide.dfci.harvard.edu/).

### Cell abundance calculation in bulk RNA-seq data

Gene expression counts from cells in TAM-CCL3, TAM-AIF1, TAM-SPP1, endothelial cells, glioma cells, oligodendrocytes and lymphocytes from the single cell dataset were used to generate the reference matrix with “CIBERSORTx” [78]. The minimum expression parameter was set to 0.25 for the reference matrix generation. Deconvolution was then performed on the TCGA and CGGA bulk RNA-seq data (fragments per kilobase per million mapped reads (FPKM)) using S-mode batch correlation and absolute mode as previously described [128].

### Survival analysis

The patients’ overall survival between different levels of TAMs and risk score, as well as patients in different Clusters were evaluated by Mantel-Cox Log-Rank tests using the “survival” (Version 3.5.3), and survival curves were visualized using Kaplan–Meier plots by the “survminer” (Version 0.4.9).

### Prognostic model construction

Consensus clustering was performed to identify molecular subtypes associated with different immune contexture-related signatures via the “ConcensusClusterPlus” (Version 1.62.0). The maximum cluster number was set to 6. Differential gene expression analysis between Cluster 1 and Cluster 2 patients was calculated using the “limma” (Version 3.54.0). 1959 differentially expressed genes with |log2FoldChange| > 0.6 and FDR < 0.05 were identified. 1311 genes with univariate Cox regression p < 0.05 were further selected for LASSO Cox regression analysis using the “glmnet” (Version 4.1.2). Finally, four genes were selected according to their variable importance rankings by the “randomforest” (Version 4.7_1.1). The four genes were weighted by relative coefficient in the multivariate Cox-PH regression and the risk score was calculated as follows: risk score = (0.013778009) × *APOBEC3C* + (0.003622016) × *EMP3* + (0.009527270) × *IGF2BP2* + (0.056328348) × *TGIF1*.

### Somatic alterations analysis

Mutation information including SNV and CNV derived from whole-exome sequencing (WES) and SNP array (Affymetrix Genome-Wide Human SNP Array 6.0) as well as corresponding clinical data of primary low grade glioma tumors were obtained from the TCGA database. The somatic variant profiles were visualized and summarized using the package “maftools” (Version 2.14.0) [129] and “GISTIC2.0” [82].

### Statistical analysis

Data were analyzed using the R software (Version: 4.1.3) for all the statistical analyses. Kaplan–Meier analysis with log-rank test was used for survival difference between groups. Statistical comparison between two groups was evaluated by two-tailed and unpaired Student’s t test. Correlation analysis was assessed by Spearman’s correlation. The relationship between two categorical variables was computed by Chi-square test. Significance was defined as p value < 0.05.

### Supplementary Information

**Additional file 1: Figure S1.** Illustration of cellular communication network between TAMs and tumor cells in the glioma microenvironment. **a** UMAP plots showing expression patterns of microglia and macrophage markers in glioma single cells. **b** Percentage of cell populations as identified in (Fig. 1a). **c** Chord plot and heatmap showing significantly interacting pathways and communication probability of the PDGF pathway between TAMs and tumor cells in the glioma TME. **d** Circle plot and violin plot displaying the ligand-receptor pair in the PDGF pathway and their expression patterns across cell clusters.

**Additional file 2: Figure S2.** Expression of marker genes in each TAM cluster within the glioma single-cell dataset and their correlation to patient survival. **a** Violin plots representing the expression levels of *CCL3L1*, *P2RY12* and *TREM2* across different clusters. **b** Kaplan-Meier curves delineating the survival based on the gene expression levels of markers associated with the three subtypes of TAMs in TCGA glioma patients. Statistical significance was assessed by two-sided log-rank test for (**b**).

**Additional file 3: Figure S3.** Expression of TFs in the TAM subsets and the validation of the TAM subsets within a mouse cohort. **a** Analysis of TIDE, T cell dysfunction and T cell exclusion scores in TCGA glioma patients with high and low marker scores for TAM-CCL3. **b** Heatmap displaying the expression of top three TFs in each cluster. **c, d** UMAP projection of 9,512 myeloid cells from non-tumor bearing, LGG and HGG mice; coloured by sample (**c**) and cell types (**d**). **e** UMAP plots showing expression patterns of *Aif1*, *Ccl3* and *Spp1* in mouse single cells. **f** UMAP plots showing the distribution of SPP1^+^TAM in non-tumor bearing, LGG and HGG mice. Statistical significance was determined by unpaired two-tailed Student’s t-test for (**a**).

**Additional file 4: Figure S4.** Deconvolution of major cell types in glioma patients using bulk RNA-seq data. **a** Relative abundance of seven cell types in glioma patients from the TCGA and CGGA datasets, estimated by the CIBERSORTx algorithm. **b** Kaplan-Meier curves of overall survival according to the proportions of TAM-AIF1 in TCGA and CGGA datasets. Statistical significance was assessed by two-sided log-rank (Mantel-Cox) test for (**b**)

**Additional file 5: Figure S5.** Workflow of the construction for the prognostic model. **a** Workflow for the establishment of the prognostic model predicting survival outcomes in glioma patients. **b** Visualization of LASSO coefficients based on results from univariate Cox regression analysis. **c** Variable importance ranking as determined by random forest algorithm. The rank is based on the mean decrease Gini value. **d, e** Univariate (**d**) and multivariate (**e**) Cox regression analyses assessing the relationship between clinical indicators, risk scores, and the overall survival of glioma patients. **f, g** Spearman correlation between risk scores and the signature score of immune checkpoint markers in the TCGA (**f**) and CGGA (**g**) glioma dataset.

**Additional file 6: Figure S6.** Somatic variation profiles of distinct glioma clusters. **a, b** The variant classification and counts, variant type and SNV class in Cluster 1 (n = 241) (**a**) and Cluster 2 (n = 266) (**b**) patients. **c, d** Detailed information of the top 20 mutated genes among Cluster 1 (**c**) and Cluster 2 (**d**) patients. The percentage and mutation class of each gene are shown on the right side. **e, f** GISTIC2.0 results plotted as gene numbers and mutated samples in altered cytobands in Cluster 1 (**e**) and Cluster 2 (**f**) patients. The size of each bubble indicating -log_10_ transformed q values.

**Additional file 7: Figure S7.** Association between *EGFR* amplification and prognostic markers. **a** Violin plot representing the differential *EGFR* mRNA levels (log_2_(TPM + 1)) in TCGA glioma patients with or without *EGFR* amplification. **b** Kaplan-Meier curve of overall survival according to *EGFR* CNV status in TCGA glioma patients. **c** UMAP plot of *EGFR* expression pattern in glioma single cells. **d** UMAP projection of 92,102 single cells from grade 4 GBM tissues, coloured by graph-based cell clusters and annotated cell types (TAM-BDM: TAM-bone marrow derived macrophage; TAM-MG: TAM-microglia). Bar chart comparing the number of cells across four different cell types between patients with normal *EGFR* and amplified *EGFR*. **e** Ridgeline plot showing the expression levels of *SPP1* and *CCL4* across four different cell types. **f**–**i** Violin plots representing differential expression levels of prognostic markers in TCGA glioma patients with or without *EGFR* amplification. Statistical significance was assessed by unpaired two-tailed Student’s t-test for (**a, f**–**i**) and by two-sided log-rank test for (**b**). ****p < 0.0001.

**Additional file 8: Table S1.** Marker genes for the seven major clusters from the two low-grade patients. **Table S2.** Details of cell type annotation by ScType. **Table S3.** Marker genes for the 13 clusters from the low-grade patients. **Table S4.** Marker genes for each TAM cluster and lymphocyte cluster from the 17 cross-grade glioma patients. **Table S5.** Patient characteristics of the TCGA cohort. **Table S6.** Patient characteristics of the CGGA cohort. **Table S7.** Signature markers of the three TAM subsets. **Table S8.** Summary of public single cell datasets used in this study.

## Data Availability

The authors confirm that the data supporting the findings of this study are available within the article and/or its supplementary materials. The codes used in this study are available on Github (https://github.com/ws2024/glioma2024).
